# Integrated metabolic profiling and transcriptome analysis of pigment accumulation in diverse petal tissues in the lily cultivar ‘Vivian’

**DOI:** 10.1186/s12870-020-02658-z

**Published:** 2020-09-29

**Authors:** Xiaojuan Yin, Xinyue Lin, Yuxuan Liu, Muhammad Irfan, Lijing Chen, Li Zhang

**Affiliations:** 1 College of Horticulture, Key Laboratory of Protected Horticulture (Ministry of Education), College of Biosciences and Biotechnology, Shenyang Agricultural University, Shenyang, 110866 Liaoning China; 2 Department of Biotechnology, University of Sargodha, Sargodha, Pakistan

**Keywords:** Anthocyanins, Cyanidin, Transcriptome sequencing, Metabolome sequencing, Scanning electron microscopy, Transporters, Transcription factors, Lily

## Abstract

**Background:**

Petals are the colorful region of many ornamental plants. Quality traits of petal color directly affect the value of ornamental plants. Although the regulatory mechanism of flower color has been widely studied in many plants, that of lily flower color is still worth further exploration.

**Results:**

In this study, the pigmentation regulatory network in different regions of the petal of lily cultivar ‘Vivian’ was analyzed through tissue structure, metabolites biosynthesis, and gene expression. We found that cell morphology of the petal in un-pigmented region differed from that in pigmented region. The cell morphology tends to flatten in un-pigmented region where the color is lighter. Moreover, high level anthocyanin was found in the pigmented regions by metabonomic analysis, especially cyanidin derivatives. However, flavanones were accumulated, contrast with anthocyanin in the un-pigmented regions of lily petal. To understand the relationship of these different metabolites and lily flower color, RNA-Seq was used to analyze the differentially expressed genes-related metabolite biosynthesis. Among these genes, the expression levels of several genes-related cyanidin derivatives biosynthesis were significantly different between the pigmented and un-pigmented regions, such as *LvMYB5, LvMYB7, LvF3’H, LvDFR, LvANS* and *Lv3GT*.

**Conclusions:**

This data will help us to further understand the regulation network of lily petal pigmentation and create different unique color species.

## Background

In ornamental plants, flower petals are a primary characteristic and the quality of the flower color directly affects the aesthetic and commercial value of plants. In plants, a variety of pigmentation patterns, such as stripes or spots, is usually the result of spatial regulation of gene expression. For example, the formation of stripes and spots in antirrhinum and phalaenopsis orchids were related to *MYB* [1, 2]. In the natural bicolor floral phenotype in petunia, the mature *CHS* mRNAs were not found in the white tissues. It indicated that the bicolor floral phenotype was caused by the spatially regulated post-transcriptional silencing of both *CHS-A* [3]. And in the previous studies, the researchers studied the pigmentation patterns in lily petals with spots and stripes. It was suggested that the *LhMYB12* regulated the formation of anthocyanin in spots and stripes [4, 5]. In this research, we wanted to clarify another pigmentation pattern in lily petal with pigmented in the top and un-pigmented in the basal.

The distribution and accumulation of colored compounds in different regions may also be related to changes in organizational structure [1, 6]. From the perspective of cytology, the epidermal cells of petals where anthocyanidin is distributed not only protect the petals structurally, but also greatly influence the formation of flower color. Light strikes the petals, part of which enters the epidermis, where it is absorbed by the petals and converted into energy. The other portion is reflected back by the different structures and tissues of the petals and through the pigment layer to produce color [7]. Therefore, the petals of different epidermal cell structures result in different proportions of incident light and reflected light, which then affects the flower color.

Studies found that the epidermal cells of petals have different shapes, which are usually pointed, conical or flat [8]. The study in *primulaceae* found that anthocyanins were found in the epidermis cell of flower, and most epidermal cells had round arched and sharp conical shape cells [9]. Furthermore, the study in the pigment distribution and epidermal cell shape of *dendrobium* species found that there were four types of epidermal cell shapes identified in *dendrobium* flowers: flat, dome, elongated dome and papillate. The epidermal cell shape of the plant affected the visual perception [10].

Additionally, plant pigments are the most important determinant of flower color. Differences in the accumulation of natural product pigments like flavonoids and carotenoids are considered the primary basis of floral pigmentation. The kind and content of such pigments are the direct cause of different petal colors [11]. Anthocyanidin accumulation usually occurs during lily flower development [12]. In higher plants, pelargonidin (brick red), cyanidin (from red to pink), and delphinidin (from blue to purple) are the most common anthocyanidins, and are all secondary metabolites in the flavonoid metabolic pathway [13].

Flavonoid biosynthesis and physiology of anthocyanins has received extensive research attention. The biosynthesis of flavonoids comes from the phenylpropanoid pathway and involves a variety of biosynthesis pathways and regulatory genes. The catalytic enzyme genes of flavonoid biosynthesis include *CHS* (chalcone synthase), *CHI* (chalcone isomerase), *F3’H* (flavanone 3′ hydroxylase), *F3′5’H* (flavanone 3’5’ hydroxylase), *FLS* (flavonol synthase), *DFR* (dihydroflavonol 4-reductase), *ANS* (anthocyanidin synthase), and *UFGT* (UDP-flavonoid glucosyl transferase). These genes synergistically regulate the synthesis of anthocyanin [14, 15]. *R2R3-MYB* [16–18], *bHLH* (basic helix-loop-helix) [19], and *WD40* (WD40 repeat protein) [20] are the main transcription factors (TFs) that regulate anthocyanin synthesis [21]. Extensive work has demonstrated the functions of these regulatory genes. For example, R2R3-MYBs in Subgroups 4, 5, 6 and 7 of *Arabidopsis thaliana* are involved in synthesis and regulation of anthocyanins and proanthocyanins in the flavonoid biosynthesis pathway [22], whereas the bHLHs in Subgroup 3 are involved in regulating the flavonoid biosynthesis pathway as TFs [23, 24]. Therefore, the formation of petal color is affected by a variety of factors, including the organizational structure of petals, secondary metabolites, and regulation of gene expression.

**Fig. 1:**
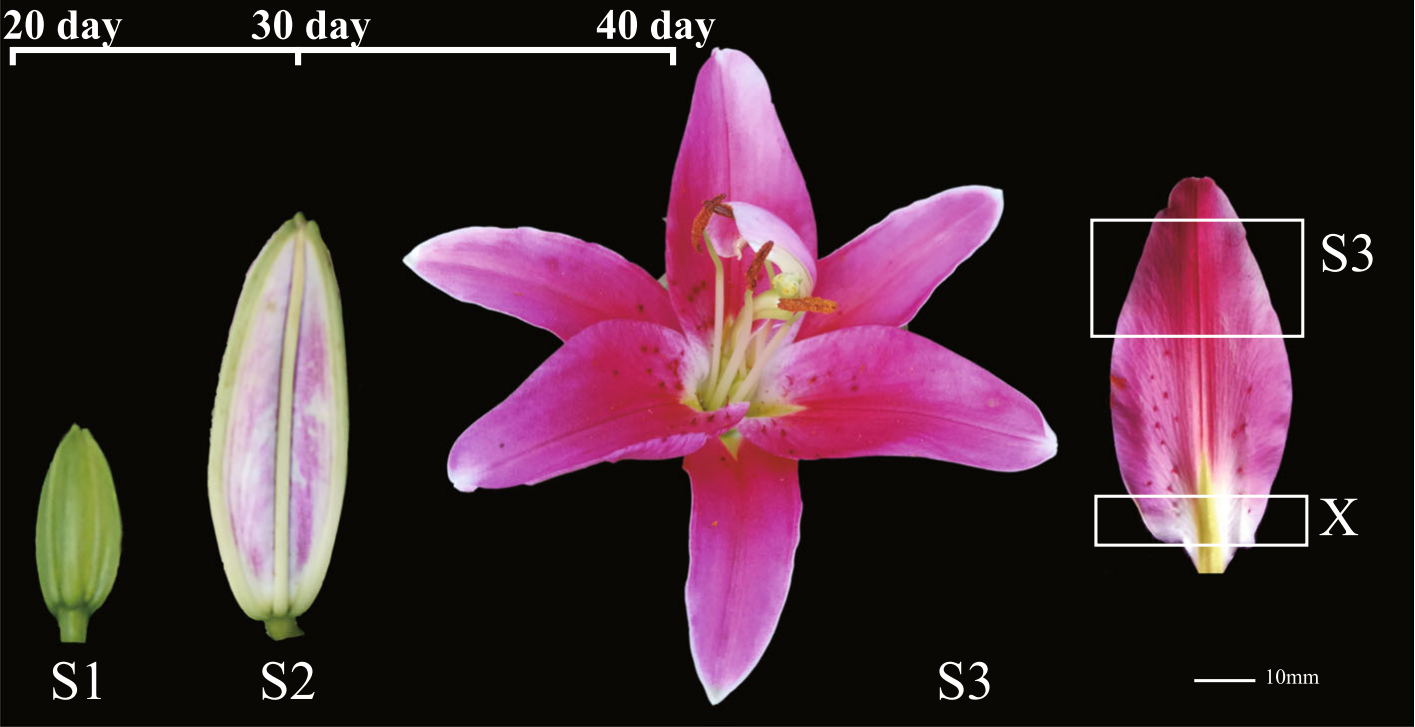
Growth and development stages of lily cultivar ‘Vivian’. S1: the bud stage, 20 days after bud formation. S2: the bud coloring stage, 30 days after bud formation. The blooming stage, 40 days after bud formation, which was divided into two regions: S3 and X. S3: the pigmented region of the petal; X: the un-pigmented region of the petal. The pictures were taken during the development of the lily flower

Lily cultivars have variable characteristics. For example, Asian hybrid lily has rich color, but lacks fragrance, while Oriental hybrid lily flowers are large and beautiful with a strong aromatic fragrance, but relatively simple coloration. Therefore, one of the goals of flower breeding is to cultivate lily with abundant flower color and floral fragrance. In addition, the spots, streaks and uneven pigment accumulation in the petals of lily can be used to form diverse lily cultivars. These formation mechanisms have also attracted extensive attention in the study of ornamental plants. Additionally, studies on the regulatory gene of flower color in lily have shown that *LhMYB12* promotes the coloring of whole petals [25, 26]; *LhMYB12-lat* determines the formation of anthocyanin in ovary [27]; *LrMYB15* regulates anthocyanin pigmentation in bud in *Lilium regale* [28]; and *LhMYB18* is involved in the formation of large anthocyanin spots [29]. The lily cultivar ‘Vivian’, which is an Oriental hybrid lily, is fragrant, bright-colored and is highly desired by consumers. In the blooming stage of ‘Vivian’, the top and bottom regions of the petal have different coloration. The top of the petal is dark pink, while the bottom of the petal is un-pigmented and white (Fig. 1).

In this study, the lily cultivar ‘Vivian’ was used to study the pigmentation regulatory network of different petal regions during the development of lily flowers. Due to the different degree of pigmentation in different regions of lily petals, we speculated that there were differences between the two-color regions in morphology of petal cells, anthocyanin metabolites and expression levels of pigmentation genes. So, the scanning electron microscopy (SEM) was performed on different regions of lily petals, which were collected from lily cultivar ‘Vivian’, ‘Table dance’, and ‘Corvara’ at the blooming stage, to observe the morphology of epidermal cells and the correlation with the color in different regions. We next sectioned the petals of ‘Vivian’ into two parts, the upper pigmented regions and the lower un-pigmented regions, then conducted high-resolution LC-MS-based metabolomics analysis to analysis the accumulation of anthocyanin biosynthetic metabolites in different regions. We then used the same sample types for RNA-seq, which enabled us to characterize the differential expression of hundreds of genes between the petal regions and between emerged and developing flowers by sampling over roughly 40 days. To screen out the functional genes attribute to petal pigmentation in the molecular level.

## Results

### Morphological differences of epidermal cells in different color regions of lily petals

**Fig. 2:**
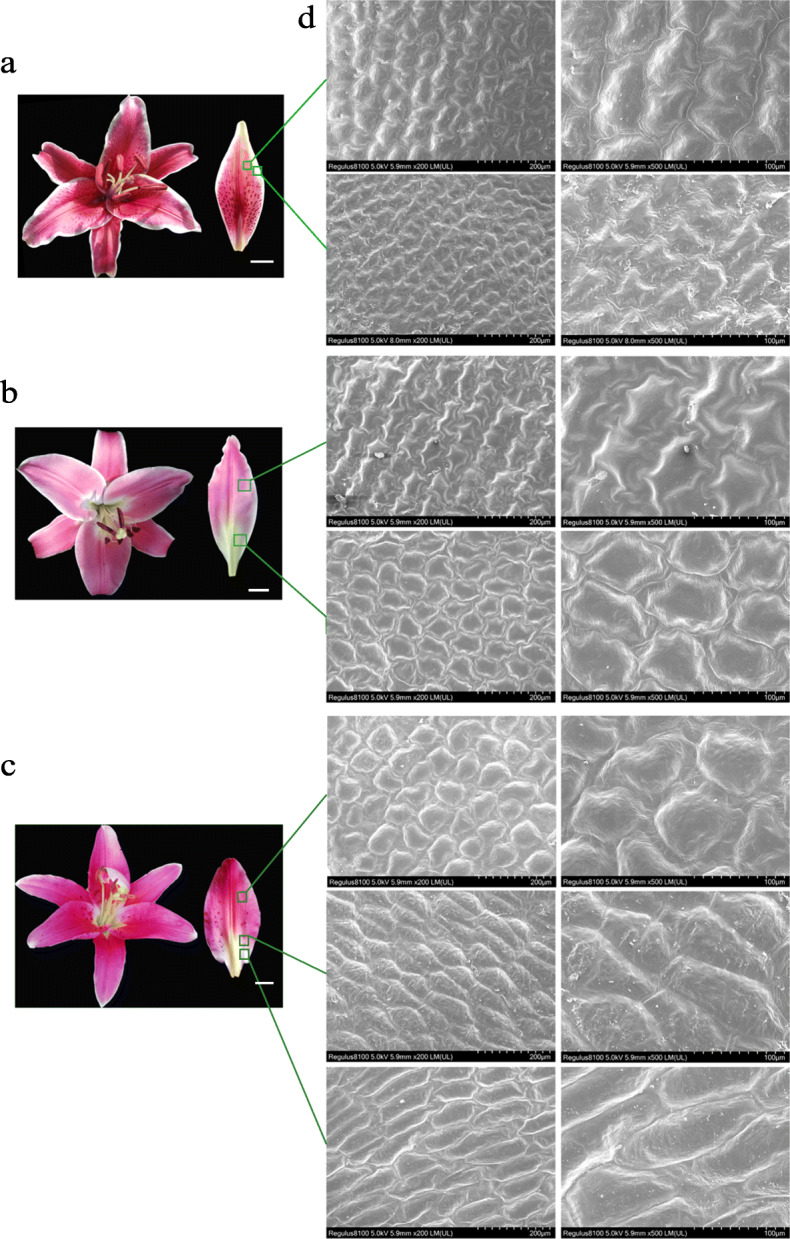
Electron microscopic observation of the epidermal cell structure of lily petals during the blooming stage. **a** Lily cultivar ‘Corvara’ flower and petal; **b** Lily cultivar ‘Table dance’ flower and petal; **c** Lily cultivar ‘Vivian’ flower and petal; Bar = 10 mm (**d**): the morphology of epidermal cells in different regions of lily petals with magnifications of 200 times (left, Bar = 200 μm) and 500 times (right, Bar = 100 μm). The samples were observed and photographed by SEM

In lily cultivars ‘Corvara’ (Fig. 2a), ‘Table dance’ (Fig. 2b), and ‘Vivian’ (Fig. 2c), the petals displayed a color gradient from pigmented to un-pigmented. Scanning electron microscopy (SEM) was used to observe the cell morphology in different regions of the petals, which were collected in the blooming stage of lily flower, in order to determine the effect of epidermal cells on petal color. We found that the cell morphology of the petal differed in the pigmented and un-pigmented regions. The morphology of epidermal cells in the pigmented region of ‘Corvara’ petals was inlaid and convex, while the morphology of epidermal cells in the un-pigmented region was inlaid and flat (Fig. 2a, d). The morphology of epidermal cells in the pigmented region of ‘Table dance’ petals was irregular and convex, while the morphology of epidermal cells in the un-pigmented region was irregular and flat (Fig. 2b, d). The morphology of epidermal cells in the pigmented region, both top and margin, of ‘Vivian’ petals was cone-shaped and convex, in the central junction region it was rhombic and flat, and in the un-pigmented region at the base it was flat (Fig. 2c, d). The morphology of epidermal cells in the white petals of lily cultivar ‘Vestaro’ was flat (Additional file 1: Figure S1).

### LC-MS was used to analyze the changes of secondary metabolites in lily cultivar ‘Vivian’

In addition to the influence of epidermal cell structure of epigenetic traits, we wanted to determine differences in the types of floral pigments accumulated in each region. With the lily cultivar ‘Vivian’, we used an LC-MS assay to profile metabolites accumulated in three sample types: bud stage petal sample (S1), a 1–2 cm region down from the top of the inner petals at 20 days after bud formation; blooming stage petals, which were divided into two parts – the pigmented petal sample (S3), a 2–3 cm region down from the top of the inner petals in the blooming stage at 40 days after bud formation, and a 1 cm region up from the base for the un-pigmented petal sample (X). Every sample had three replicates from different triennial plants. In total, 652 distinct ion peaks were detected amongst the samples by LC–MS.

Subsequent analysis of the metabolites accumulated in the pigmented petal samples (S3) vs. un-pigmented petal samples (X) identified 204 significantly differentially accumulated metabolites based on a VIP > 1 threshold in the PLS-DA model and a *P* < 0.05 significance threshold in Student’s *t*-test analysis (after FDR correction). KEGG analysis showed that the significantly enriched pathways of the differentially accumulated metabolites in X vs. S3 were flavonoid biosynthesis (Additional file 2: Figure S2). We selected 23 differentially accumulated metabolites about this pathway to analysis, which include coumarins, hydroxycinnamoyl derivatives, flavanone, flavonol and anthocyanins metabolites (Table 1).

**Table 1:** Metabolites associated with anthocyanin biosynthesis in S3 compared to X

Index	Compounds	Class	LogFC
pme3413	Coumarin	Coumarins	1.04
pme3245	Medicarpin	Hydroxycinnamoyl derivatives	1.71
pme0305	Ferulic acid	Hydroxycinnamoyl derivatives	2.13
pme1424	Coniferylaldehyde	Hydroxycinnamoyl derivatives	2.63
pme1637	Coniferyl alcohol	Hydroxycinnamoyl derivatives	3.81
pme0424	trans-cinnamaldehyde	Hydroxycinnamoyl derivatives	1.16
pme1436	p-Coumaric acid	Hydroxycinnamoyl derivatives	2.03
pme0002	Hesperetin 7-O-neohesperidoside (Neohesperidin)	Flavanone	1.10
pme0371	Naringenin 7-O-glucoside (Prunin)	Flavanone	1.11
pme1622	Kaempferol 3-O-glucoside (Astragalin)	Flavonol	5.28
pme0197	Quercetin 3-O-rutinoside (Rutin)	Flavonol	1.12
pme3211	Quercetin 3-O-glucoside (Isotrifoliin)	Flavonol	4.74
pme3267	Kaempferol 3-O-galactoside (Trifolin)	Flavonol	1.94
pme3297	Kaempferol 3-O-rhamnoside (Kaempferin)	Flavonol	3.35
pme1521	Dihydroquercetin (Taxifolin)	Flavonol	2.05
pme0199	Quercetin	Flavonol	3.50
pme0196	Kaempferol	Flavonol	2.77
pmb0550	Cyanidin 3-O-glucoside (Kuromanin)	Anthocyanins	2.95
pme1773	Cyanidin 3-O-rutinoside (Keracyanin)	Anthocyanins	6.28
pme3609	Cyanidin	Anthocyanins	3.30
pme2321	Hesperetin	Flavanone	−18.97
pme3461	Homoeriodictyol	Flavanone	−16.99
pme0442	Delphinidin	Anthocyanins	−2.27

**Fig. 3:**
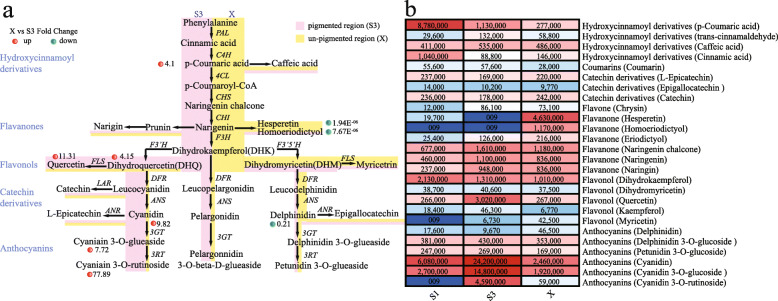
Flavonoid biosynthesis pathway and the differential metabolites of different petal regions in lily cultivar ‘Vivian’. **a** Flavonoid biosynthesis pathway in lily cultivar ‘Vivian’. The red dots represent the up-regulated metabolites, while the green dots represent the down-regulated metabolites in S3 compared to X. The width of pink and yellow represented the relative substance content of S3 and X, respectively. **b** The differential metabolites in lily cultivar ‘Vivian’. Each colored cell represents the average value of each metabolites

We also conducted a combined analysis of the flavonoid biosynthesis pathway (Fig. 3a) and differential metabolites (Table 1). The substances, cinnamic, p-coumaric and coumaroyl-CoA acids are the beginning of the flavonoid biosynthesis pathway. We found that the contents of p-coumaric acid in the pigmented petals (S3) were significantly higher than that in the un-pigmented petals (X). P-coumaric acid substrate was catalyzed to produce coumaroyl-CoA and caffeic acid, but there was no significant difference between the two substances in different regions of the petal. Naringenin chalcone and naringenin were catalyzed to produce flavanone and flavonol materials. Dihydroquercetin (DHQ), which belongs to the flavonol class, is a necessary substrate for the synthesis of cyanidin, and the content of DHQ in S3 was higher than X. The derivatives, cyanidin, cyanidin 3-O-glucoside (kuromanin), and cyanidin 3-O-rutinoside (keracyanin) were respectively up-regulated by 9.8-, 7.7-, and 77-fold in S3 compared to X in ‘Vivian’. At the same time, several down-regulated metabolites were found in S3 compared to X, including flavanones of hesperetin, homoeriodictyol (down-regulated by 1.94E^− 06^- and 7.76E^− 06^-fold in X compared to S3, respectively) and anthocyanins of delphinidin (down-regulated by 0.214-fold in X compared to S3).

The results showed that the contents of hesperetin and homoeriodictyol in the same petal were significantly higher in the un-pigmented region (X) than in the pigmented region (S3). In contrast, the content of cyanidin derivatives in the un-pigmented region (X) was significantly lower than that in the pigmented region (S3). Although the content of delphinidin in X was relatively high, there was no significant difference between petunidin-3-glueaside in X and S3. Overall, in the determination of metabolites in the anthocyanin synthesis pathway, the major anthocyanins in ‘Vivian’ were the cyanidin derivatives.

We also analyzed the metabolites accumulated in S1 vs. S3 and identified 179 significantly differentially accumulated metabolites based on the same standard as above. KEGG analysis showed that the significantly enriched pathways of the differentially accumulated metabolites were flavone and flavonol biosynthesis (Additional file 3: Figure S3). In total, 23 differentially accumulated metabolites (Table 2) were screened to analysis. As the initial substrates, we found that cinnamic acid and p-coumaric acid were higher in ‘Vivian’ in S1 than S3, while the content of hesperidin in S1 was also high compared to S3. The contents of cyanidin 3-O-glucoside, cyanidin 3-O-rutinoside, and cyanidin were higher in S3 than S1 (Table 2). In summary, LC-MS analysis showed that, with reduced content of cinnamic acid, p-coumaric acid and hesperidin in the early stage, the products of other flavonoid and anthocyanins were accumulated continuously in the process of flower pigmentation.

**Table 2:** Metabolites associated with anthocyanin biosynthesis in S3 compared to S1

Index	Compounds	Class	LogFC
pme0305	Ferulic acid	Hydroxycinnamoyl derivatives	1.73
pme0424	trans-cinnamaldehyde	Hydroxycinnamoyl derivatives	2.15
pme1424	Coniferylaldehyde	Hydroxycinnamoyl derivatives	3.94
pme1637	Coniferyl alcohol	Hydroxycinnamoyl derivatives	14.14
pme2306	Eugenol	Hydroxycinnamoyl derivatives	13.71
pme3245	Medicarpin	Hydroxycinnamoyl derivatives	1.94
pme0330	Naringenin 7-O-neohesperidoside (Naringin)	Flavanone	2.00
pme1580	Eriodictyol	Flavanone	2.31
pme2979	Pinocembrin (Dihydrochrysin)	Flavanone	15.05
pme0199	Quercetin	Flavonol	3.50
pme1478	Myricetin	Flavonol	9.55
pme1521	Dihydroquercetin (Taxifolin)	Flavonol	3.13
pme1622	Kaempferol 3-O-glucoside (Astragalin)	Flavonol	2.42
pme3211	Quercetin 3-O-glucoside (Isotrifoliin)	Flavonol	5.82
pme3401	Syringetin	Flavonol	10.86
pmb0550	Cyanidin 3-O-glucoside (Kuromanin)	Anthocyanins	2.46
pme1773	Cyanidin 3-O-rutinoside (Keracyanin)	Anthocyanins	18.96
pme3609	Cyanidin	Anthocyanins	1.99
pmb0142	Caffeic aldehyde	Hydroxycinnamoyl derivatives	−1.46
pme0300	Cinnamic acid	Hydroxycinnamoyl derivatives	−3.55
pme1436	p-Coumaric acid	Hydroxycinnamoyl derivatives	−2.95
pme1698	Sinapic acid	Hydroxycinnamoyl derivatives	−2.41
pme2321	Hesperetin	Flavanone	−11.10

In this study, metabolites of lily petals (Additional file 8: Table S1) in different regions or stages were detected using the widely-targeted metabolomics method. A variety of anthocyanins have been detected in the petals of Lily cultivar ‘Vivian’. There were 6 anthocyanins can be annotated into the KEGG pathway, including cyanidin 3-O-glucoside, delphinidin, delphinidin 3-O-glucoside, cyanidin 3-O-rutinoside, petunidin 3-O-glucoside, cyanidin. According to the differential metabolite analysis (Fig. 3b), it is found that petunidin 3-O-glucoside and delphinidin 3-O-glucoside show no significant difference in the sample of S1, S3 and X. And the content of delphinidin is the lowest in S3, in which stage the petals are deeply colored, but up-regulated in X. This indicated that delphinidin and petunidin were not the main metabolite affected coloring. While, the cyanidin derivatives had significant difference in different samples, and the trend of the metabolite content were positively correlated with anthocyanin accumulation. As expected, the main pigment substance of ‘Vivian’ is cyanidin derivative.

### RNA-seq was used to screen the functional genes involved in lily flower pigmentation

#### Transcriptome analysis: functional annotation and classification of unigenes

The differential accumulation of metabolites in the flavonoid biosynthesis pathway is usually due to differential expression of related genes. In order to screen the genes related to color formation, transcriptome sequencing was performed on four types of lily petal samples. Three of the samples – S1, S3, and X – were identical to the metabolome samples. We added a fourth sample, the coloring bud petal samples (S2), a 2–3 cm region down from the top of the inner petals in the coloring bud stage at 30 days after bud formation. Every sample had three replicates from different triennial plants.

The clean data generated from the library by Illumina Hiseq Sequencing (Additional file 9: Table S2). In total, 125,535 unigenes were assembled, with a mean length of 563 bp (N50 length of 896 bp) (Additional file 10: Table S3). The gene functions were annotated based on five databases (Additional file 11: Table S4). These results indicated that the RNA-Seq data of Lily cultivar ‘Vivian’ petals were usable in this study.

GO analysis showed that 15,520 unigenes annotation can be enriched, and divided into three categories: biological processes, cell components, molecular function. In molecular function, the binding (7686 Unigene, 49.5%) was one of the biggest groups, catalytic activity (6895 Unigene, 44.4%) was the next. Cellular process (7449 Unigene, 48.0%) and metabolic process (7406 Unigene, 47.7%) were the two largest categories in biological processes (Additional file 4: Figure S4).

#### The differentially expressed genes in flowering development

To find the functional genes related to the flavonoid biosynthesis during flower development, we analyzed the samples of S1 (bud petals), S2 (coloring bud petals), and S3 (pigmented petals). All three samples were collected from the region without veins of the petals in flowers at different developmental stages. The differentially expressed genes (DEGs) in different stages of flower development were statistically analyzed using |log2FoldChange| > 1, *P*-value< 0.05. There were 4622 DEGs in S1 vs. S3 with 2455 up-regulated genes and 2167 down-regulated genes; 3636 DEGs in S2 vs. S3 with 2191 up-regulated genes and 1445 down-regulated genes; and 2601 DEGs in S1 vs. S2 with 1139 up-regulated genes and 1462 down-regulated genes (Additional file 5: Figure S5a).

KEGG analysis showed that the significantly enriched pathways of the DEGs were involved in plant hormone signal transduction, starch and sucrose metabolism (Additional file 5: Figure S5b-d). The pigmented process of lily petal is also the process of flower development from bud to maturity. Previous studies have shown that the plant hormone signal transduction, starch and sucrose metabolism were closely related to the plant growth and development [30–32]. Therefore, it may perform a similar function in the flower development of lily.

#### The functional genes related to flavonoid and anthocyanin biosynthesis

**Fig. 4:**
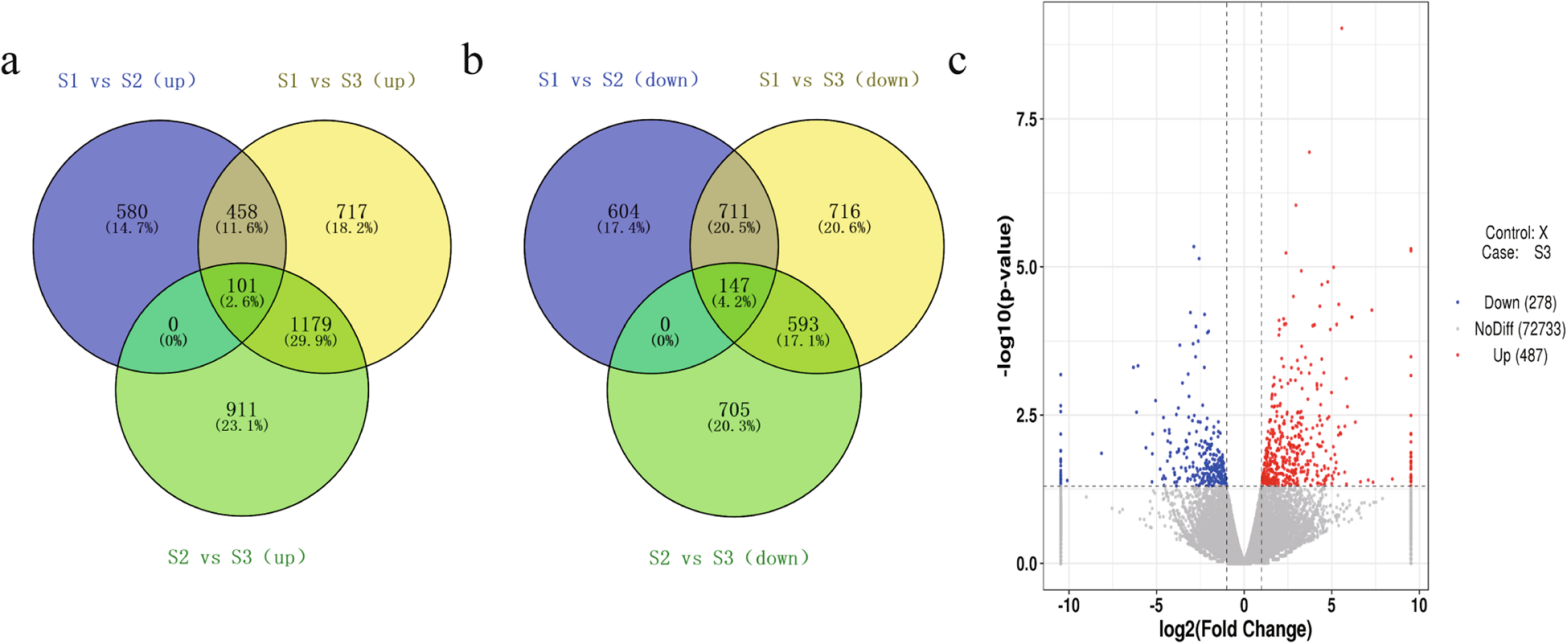
The differentially expressed genes (DEGs) in different stages of flower development. Venn diagram of DEGs in flower development. **a** Venn diagram of the up-regulated DEGs in flower development. **b** Venn diagram of the down-regulated DEGs in flower development. **c** Volcano Plot of DEGs between pigmented and un-pigmented petals

According to the differentially expressed genes (DEGs) from bud stage (S1) to coloring stage (S2) or to blooming stage (S3), in which process the petal was coloring, there were 559 DEGs up-regulated (Fig. 4a) and 858 DEGs down-regulated (Fig. 4b). In the result of LC-MS, different metabolites in different regions of lily petals have significant differences in the pathway of flavonoids biosynthesis, which was related to flower pigmentation. In order to analyze the regulation network about lily flower color, the genes related to flavonoid biosynthesis were screened from up-regulated and down-regulated DEGs for analysis.

**Fig. 5:**
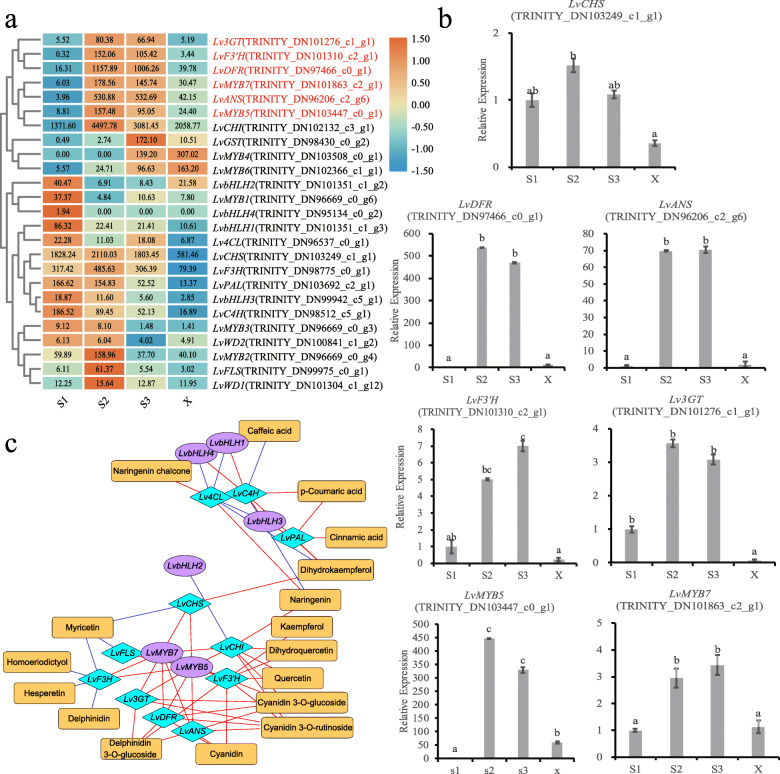
Correlation mapping between intermediate metabolites and gene expression in lily cultivar ‘Vivian’ petals. **a** Heatmap of functional gene expression. Each colored cell represents the average log_2_(FPKM) value of each pathway gene, then performed rows cluster. **b** Relative expression levels of genes during flower development of ‘Vivian’. Relative expression analysis of genes in bud petals (S1), coloring petals (S2), pigmented petals (S3), and un-pigmented petals (X) using the 2-ΔΔCt method. Data are means (±SD) of three biological replicates per variety. Different letters indicate statistically significant differences among the samples. **c** The regulatory network between secondary metabolites and related genes in the anthocyanin synthesis pathway; orange rectangular nodes represent metabolites; purple oval nodes represent TF genes; blue rhombic nodes represent structural genes; blue lines represent repression; and red lines represent activation

We found that the expression levels of early structural genes *LvPAL* (TRINITY_DN103692_c2_g1) and *LvC4H* (TRINITY_DN98512_c5_g1) were down-regulated during flower development from S1 (bud stage) to S3 (blooming stage) (Fig. 5a). The results were consistent with cinnamic acid and p-coumaric acid (Table 2). The FPKM of *LvCHI* was relatively high during all three stages (the mean FPKM was greater than 1000), but the FPKM of *LvCHS* in the three stages was not significantly different. Therefore, the high expression of early structural genes promotes the encoding of enzymes to catalyze substrates, providing more flavonoid and flavonol substrates for the formation of anthocyanins.

The late regulation genes *LvANS* (TRINITY_DN96206_c2_g6, 283.8-fold), *LvF3’H* (TRINITY_DN101310_c2_g1, 226.4 -fold), *LvDFR* (TRINITY_DN97466_c0_g1, 79.8 -fold), and *Lv3GT* (TRINITY_DN101276_c1_g1, 32-fold) were up-regulated in S2 compared to S1. The genes *LvANS, LvF3’H, LvDFR*, and *Lv3GT* were up-regulated by 99-, 88-, 32- and 6-fold in S3 compared to S1, respectively, with significant differences in expression level (Fig. 5a). All of these genes may function in determining pigmentation during flower development.

In summary, the expression of *LvANS, LvF3’H, LvDFR* and *Lv3GT* were consistent with the formation and accumulation trend of anthocyanin during flower development. The expression levels of these genes were significantly higher in S2 or S3, and significantly lower in S1 and X. This pattern may synergistically promote the synthesis and accumulation of anthocyanins in ‘Vivian’ petals.

Transcription factors (TFs) can synergistically regulate gene expression in the flavonoid biosynthesis pathway, so we further analyzed TFs. We compared the Pfam model of *MYB* and *bHLH* in the lily transcriptome data, and screened out the genes containing MYB and HLH motif. We analyzed all genes annotated as *MYB* and *bHLH*, which differed significantly in S1 vs. S2, S2 vs. S3, and S1 vs. S3, and constructed separate evolutionary trees with *Arabidopsis MYB* and *bHLH* (Additional file 6: Figure S6a, c), to further analyze the differentially expressed TF genes (Additional file 6: Figure S6b, d).

Two *MYBs*, *LvMYB7* (TRINITY_DN101863_c2_g1) and *LvMYB5* (TRINITY_DN103447_c0_g1), clustered with subgroup 6 of *Arabidopsis thaliana* MYB gene family (*AtMYB75*, *AtMYB90*, *AtMYB113* and *AtMYB114*), which can regulate the biosynthesis of anthocyanins in the late developmental stages [33], with higher FPKM and significantly different gene expression levels during flower development. Moreover, its expression level was higher in S2 or S3, lower in S1 and X, and changed significantly. The III group of *A. thaliana* bHLH gene family is involved in regulating the synthesis of flavonoids [23]. Analysis of DEGs identified four lily *LvbHLH* (TRINITY_DN101351_c1_g2, TRINITY_DN101351_c1_g3, TRINITY_DN99942_c5_g1 and TRINITY_DN95134_c0_g2), that clustered with *Arabidopsis thaliana* bHLH gene family in III group with significant differences in expression during flower development. However, all four genes were downregulated during flower development (Fig. 5a).

In the screen analysis of *WD*, the WD pfam model was used to screen out *WD* in the unigene. Then these genes were constructed into evolutionary trees with *WD* that related to anthocyanin biosynthesis pathway identified in other plants (Additional file 7: Figure S7). ML method in MAGA was used to construct the evolutionary trees. There were found that two genes, *LvWD1* (TRINITY_DN100841_c1_g2) and *LvWD2* (TRINITY_DN101304_c1_g1), were clustered with *WD*, which related to anthocyanin biosynthesis pathways. However, there was no significant difference in expression level of these genes (Fig. 5a).

In summary, the genes with high expression levels in S2 or S3, low expression levels in S1 and X with significant changes include *LvANS, LvF3’H, LvDFR, Lv3GT, LvMYB5* and *LvMYB7*, which are consistent with the formation and accumulation trend of anthocyanins during flower development. These genes may synergistically promote the synthesis of anthocyanins in the petals of lily.

#### Identification of flavonoid biosynthesis genes that affect petal pigmentation in different regions

In order to explore the regulatory network of petal pigmentation in different regions, we analyzed the transcriptome data of pigmented petals (S3) and un-pigmented petals (X) (Fig. 4c). There were 765 DEGs (false discovery rate [FDR] < 0.05) with 487 up-regulated genes and 278 down-regulated genes (|log2FoldChange| > 1, *P*-value< 0.05).

Based on the functional genes related to flavonoid biosynthesis in flowering development, we screened out the genes that affect pigmentation in the pigmented and un-pigmented petals. The expression levels of *LvF3’H, LvDFR, LvANS, Lv3GT*, *LvMYB7* and *LvMYB5* were higher in the pigmented petals than in un-pigmented petals (Fig. 3).

We selected seven DEGs and analyzed their expression levels in bud petal samples (S1), coloring petal samples (S2), pigmented petal samples (S3), and un-pigmented petal samples (X) using qRT-PCR (see appendix for primers), in order to validate the RNA-Seq results (Fig. 5b). And then we used Pearson correlation to identify correlations (Additional file 12: Table S5). The results of the qRT-PCR were consistent with the transcriptome data. Indicating that our transcriptome results were reliable for further studies. We found that the expression of genes, *LvF3’H, LvDFR, LvANS, Lv3GT*, *LvMYB7* and *LvMYB5* were higher in S2 or S3, with no significantly difference, and lower in S1 and X. They were also significantly higher in S3 than X. While the expression of *LvCHS* was no significantly differentially expressed.

#### Correlations between intermediate metabolites and expression of key genes

In order to test the relationship between intermediate metabolites and gene expression, we used Pearson correlation to identify correlations, and constructed the correlation network diagram with Cytoscape (Fig. 5c).

Correlation analysis showed that the expression levels of *LvMYB5* and *LvMYB7* were highly correlated with the expression levels of *LvF3’H, LvDFR, LvANS* and *Lv3GT* (Fig. 5c), which are the major late structural genes in the anthocyanin biosynthesis pathway, with high expression levels in S2 and S3 (Fig. 5a). However, the four *bHLH* of lily had high correlations with the early structural genes, which are related to the synthesis of cinnamic acid derivatives at the early stage of the anthocyanin synthesis pathway (Fig. 5c), with a similar expression trend with the cinnamic acid derivatives (Fig. 5a).

#### The sequence analysis of LvMYB5 and LvMBY7

To determine the characterization of TF MYB we screened out, a phylogenetic analysis and homologous sequence alignment were carried out using deduced amino acid sequences and other published anthocyanin-related genes amino acid sequences.

**Fig. 6:**
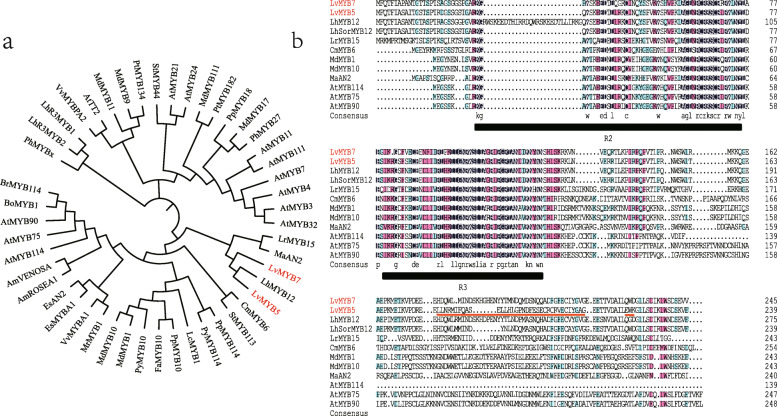
Phylogenetic analyses of *LvMYB5* and *LvMYB7* in plants. **a** Phylogenetic tree of LvMYB5, LvMYB7 amino acid sequences and other published anthocyanin-related MYBs. The GenBank accession numbers are: LhSorMYB12 (BAJ22983.1), LhMYB12 (BAO04193.1), LrMYB15 (BAU29929.1), AtMYB75/PAP1 (NP_176057.1), AtMYB90 (NP_176813.1), AtMYB114 (NP_001321376.1), MdMYB1 (ADQ27443.1), MdMYB10 (ACQ45201.1), PpMYB10 (ADK73605.1), PpMYB114 (XP_020420992.1), PyMYB114 (ASY06612.1), LcMYB1 (APP94121.1), CmMYB6 (AKP06190.1), PhMYBx (AHX24371.1), FaMYB10 (ABX79947), MrMYB1 (ADG21957), EsMYBA1 (AGT39060), EsAN2 (ALO24363), StMYB113 (ALA13584), AmROSEA1 (ABB83826), AmVENOSA (ABB83828), BоMYB1 (ADP76649), BrMYB114 (AIP94022), MaAN2 (ASF20090), VvMYBPA2 (ACK56131), PtMYB134 (ACR83705), AtTT2 (OA091653), PyMYB10 (ADN52330). **b** Multiple alignments of deduced amino acid sequences of LvMYB with other anthocyanins-related MYBs. Black lines indicate R2 and R3 domain in MYB family. Red line indicates the difference of amino acid sequence in C-terminal. The tree was constructed with the ML method (1000 replications of bootstrap test) using the MEGA5.0 program

Anthocyanin-related MYB evolutionary trees showed that the amino acid sequences of LvMYB5 and LvMYB7 had high homology (Fig. 6a), and were closely related to other published anthocyanin-related MYBs*,* which were promoting pigmentation [34–40]. The results show that LvMYB5 and LvMYB7 were clustered together with LhMYB12 of lily first. But, the homologous sequence alignment of LvMYB5, LhSorMYB12 and LhMYB12 amino acid sequences showed a high similarity at the N-terminal and several differences at the C-terminal (Fig. 6b). There was an obvious insertion of amino acids in the R2 region of LhMYB12, which differed from the sequences of LvMYB5 and LhSorMYB12. Additionally, LhSorMYB12 and LvMYB5 had high similarity in the conserved region of N-terminal, but a great difference of amino acid sequence in C-terminal (red line in Fig. 6b).

Secondly, two LvMYBs were clustered with MaAN2 of *grape hyacinth*. It was indicated that they have similar functions. MaAN2 belong to AN2 subgroup of MYB family. The accumulation of anthocyanin in *grape hyacinth* was positively correlated with the expression of MaAN2. Double luciferase assay showed that when MaAN2 was co-transformed with *Arabidopsis thaliana* AtTT8, it strongly activated the promoters of MaDFR and MaANS. The ectopic expression of MaAN2 produced a distinct red color in transgenic tobacco leaves and petals [41].

Finally, these genes were grouped with the MYB that activate anthocyanin biosynthesis in other plants, such as AtMYB90, AtMYB75 and AtMYB114 in *Arabidopsis thaliana* [23], MdMYB1 and MdMBY10 in *malus* [18, 42] and CmMYB6 in *chrysanthemum* [37]. The differences in transcript levels of *MdMYB1*, which encoded a key regulator of anthocyanin biosynthesis, controlled the anthocyanin content and color in fruit skin [43]. Over-expression of *MdMYB10* in apple generated a strong phenotype, with highly pigmented plants [42]. The transcriptional activation of *PpMYB10* resulted the anthocyanin pigmentation in tobacco [34]. The highly homology indicated that the function of these MYB were similar. In addition, LvMYB5 and LvMYB7 had the general characteristics of R2R3-MYB gene family [44], and contained R2 and R3 domains (Fig. 6b). Therefore, *LvMYB5* and *LvMYB7* were similar to other anthocyanin-related genes, which may play an important role in promoting petal pigmentation.

## Discussion

In this research we found that the morphology of epidermal cells in the region with different color was different. Then, the metabolomics analysis of upper pigmented regions and the lower un-pigmented regions were analysised. In un-pigmented regions, the metabolites of flavonoid biosynthesis pathway were mainly flowed to flavanones. While, in pigmented regions, the mainly metabolites were flowed to anthocyanin. This result has not been clarified in previous studies on lily pigmentation. Finally, we obtained two *MYB* genes, *LvMYB5* and *LvMYB7*, which had different expression levels in the pigmented and un-pigmented regions. The correlation analysis showed that *LvMYB5* and *LvMYB7* may be the main influencing factors for the coloring mode in petal different regions. It is worth to further study.

### The color of different regions of lily petal were positively correlated to the morphology of epidermal cell and the accumulation of anthocyanidins

Many studies found that the epidermal cells on the flower petals of most plants showed a distinctive conical shape. The morphology of these conical petal epidermal cells affected the light focusing and played an important role in pollinator attraction [45–47]. In this research, we found that the different regions of lily petals showed the similar morphological differences with *primulaceae* and *dendrobium* [9, 10]. The cell morphology of different regions of petal in lily showed significantly difference, namely, the darker part at the top was conical, but in the lighter part at the bottom tended to be flat. This different epidermal cell structures in petals resulted in different proportions of incident light and reflected light, which then affected flower color. For example, conical cells improved the proportion of the incident light on epithelial cells, which enhanced the light absorption by pigments, thereby leading to darkened flower color and enhanced color saturation. However, flat cells reflected more incident light, leading to lighter flower color [7]. The similar results were found in *Antirrhinum majus* [48–50].

Here, we also found that anthocyanin derivatives were little accumulated in the un-pigmented region of petal, but contrast in the pigmented region of petal in lily. Moreover, there was no visible color in the un-pigmented region. There are two reasons, one is the less pigment substances in the cells, which is insufficient for color rendering, or it may be influenced by the flattened epidermal cells on visible color. It suggests that anthocyanin biosynthesis might be involved in regulating the color formation of the pigmented region of petal in lily. However, this regulation might relate the cell morphology of the pigmented region. For example, conical epidermal cells contained significantly more anthocyanin and other flavonoids than flat epidermal cells in *Antirrhinum* petals [51]. In transgenic *Arabidopsis* plants, overexpression of *PsMYB114L* and of *PsMYB12L* caused a significantly higher accumulation of anthocyanins, resulting in purple-red leaves [52]. In *Phalaenopsis* orchids, *PeMYB11*-silencing resulted in the loss of anthocyanin content with flower colors fading from dark-purple to pink [2]. So, anthocyanin substances were closely related to the color of the plant.

The color of different regions of lily petal were closely related to the morphology of epidermal cell and the accumulation of anthocyanidins, which may be a part of influence. But we can’t deny the intrinsic cellular developmental influences. The difference in cell morphology may involve many aspects, which may be the result of the combined action, such as the hormone, light, pH, substance accumulation in the cell and other factors [53]. At the molecular level, *MIXTA* gene encodes a MYB-related protein, and participates in the transcriptional control of epidermal cell shape [48]. Research finding that *ROPs* (Plant RhoGTPases) involve in diverse aspects during plant development. It was required for the final shape formation of conical cells [54]. In addition, *SPK1* (SPIKE1), *ARP2/3* (the actin-related protein-2/3), *RIC1/4* (ROP-interactive CRIB motif-containing protein 1/4), *CRP1* (constitutive expresser of pathogenesis related genes 1), *PID* (PINOID), *ARF* (auxin response factor) and other genes are likely to be involved in the plant cell morphogenesis through hormone signaling pathways, then affect the formation of conical and flat shapes of plant cells [55–59]. Based on the research, we preliminarily screened relevant genes related to cell morphogenesis (Additional file 14: Figure S8). Moreover, the expression level of some related genes, such as *ROP, RAPC2, CRP* and *RIC*, were significantly different between pigment and un-pigment petals. Next, we will conduct detailed analysis and verification of these genes in order to describe this part with complete experimental data.

### Flavanones and anthocyanins were significantly different in different regions of lily petal

Flower development and pigmentation are closely related to flavonoid biosynthesis in most plants [60, 61]. Flavonols, flavones and cinnamic acid derivatives (CADs) in higher plants are colorless flavonoids or phenylpropyl compounds, which have the property of absorbing ultraviolet rays and have co-pigmentation with anthocyanins in flower organs [15, 62, 63]. Pelargonidin, delphinidin, and cyanidin are the primary anthocyanidins in plants. Cyanidin derivatives have been identified as being the major anthocyanins present in the skin of red apples, and also in the purplish-red, bronze, and pink chrysanthemum inflorescences [64, 65]. Lily is rich in flower color, but the metabolite composition of the flower color, and the relationship between metabolites and the flower color in different regions of petal, require further study.

Through the comparison of metabolites between the un-pigmented petals (X) and the pigmented petals (S3) of lily at the blooming stage, it can be concluded that most secondary metabolites related to the synthesis of anthocyanin are up-regulated, except for delphinidin, hesperetin and homoeriodictyol (Table 1). Delphinidin was down-regulated in S3 compared to X, indicating that the content was higher in X than in S3. However, the final content of petunidin 3-glucoside was not significantly different. It may be that the content of petunidin 3-glucoside in both regions were relatively lower, it was not enough to present a significant difference in color.

In this study, we detected many hydroxycinnamoyl derivatives, including caffeic acid and cinnamic acid, as well as flavanone, flavonol, and anthocyanins (Tables 1, 2). A large number of cinnamic acid derivatives (CADs), including caffeic acid, were detected in Asian hybrid lily, but flavones or flavonols were not detected [5, 66]. In X compared to S3, hesperetin and homoeriodictyol were down-regulated by 1.94E^− 06^ and 7.67E^− 06^-fold, with large accumulation of flavanones and downregulation of anthocyanin metabolites in un-pigmented regions (Fig. 3). Based on the anthocyanin biosynthesis pathway (Fig. 3), naringenin is the common substrate of anthocyanin, hesperetin and homoeriodictyol. This observation suggests competition for naringenin, which occurs at a branching point of the anthocyanin and flavanone biosynthesis pathways. Similar findings have been found in *petunia*, Saito et al. [67] found that in *petunia* flowers with red edges and a white center, increased flavonol products in the center of *petunia* petals suppressed the accumulated anthocyanins. Therefore, the difference of flavanone and anthocyanin substance content in different regions of lily petals leads to the different flower color in different regions. Reduction in anthocyanin levels, resulting in the formation of the un-pigmented area in the basal petal, is at least in part attributed to the accumulation of flavanone.

### *LvMYB5* and *LvMYB7* are synergistically regulators of anthocyanin accumulation in different regions of petal

The flavonoid biosynthesis pathway is a conserved network in plants, whose regulation is maintained through the expression of structural and regulatory biosynthetic genes [68]. The structural genes can be divided into early biosynthetic genes (EBGs), such as *CHS, CHI*, and *F3H,* and late biosynthetic genes (LBGs), such as *DFR, ANS,* and *UFGT*. These genes are usually regulated by *MYB* or the *MYB-bHLH-WD40* (*MBW*) complex [69, 70]. Here, we discussed which genes regulate the formation of flower color in different regions of petals in ‘Vivian’.

The expression level of EBGs in lily, such as *CHS*, was relatively high at all stages of flower development without significant differences from the perspective of metabolites. The formation mechanism was different with some bicolored flowers. The star and marginal picotee (white margin, pigmented center) patterns in petunias were caused by PTGS of *CHS-A* in the white petal regions, rather than changes in the expression of anthocyanin biosynthesis genes [3, 71]. *CHS* has been shown to play an important role in anthocyanin biosynthesis in different species, and the presence of white flowers in many plants was driven by their lack of expression [3, 72–74]. However, there was no significant difference in the expression of EBGs in lily, and there was no significant difference in the content of Naringenin chalcone. Therefore, lily is different from *petunia* in the formation mechanism of bicolor flower.

At the blooming stage of ‘Vivian’, there were significant differences in expression levels of LBGs, *LvF3’H, LvDFR, LvANS* and *Lv3GT*, between pigmented and un-pigmented petal regions, resulting in different floral pigment content and different colors in the two parts. This indicated that changes in the expression of LBGs affect the formation of different colors in flower. The expression patterns of some flower-color variation in plants were similar with ‘Vivian’. Differences in LBG expression caused color differences in bicolor peony “Shima Nishiki” [75], pink *camellia* [76], and yellow/orange variation in *Gentiana* [77]. Therefore, the expression of LBGs can affect the pigmentation in these plants.

Color patterns in angiosperm flowers are produced by spatially and temporally restricted deposition of pigments. Transcription factors (TFs) can regulate the expression of structure genes related to flower color [4]. *LvMYB5* and *LvMYB7*, two R2R3-MYB screened in this study, were closely related to anthocyanin synthesis, and it could be seen from qRT-PCR data that the expression levels of these genes were significantly different in different regions of petals. According to the network analysis of metabolites and functional genes, the expressions of *LvMYB5* and *LvMYB7* were highly correlated with the expressions of LBGs, *LvF3’H, LvDFR, LvANS and Lv3GT* in the anthocyanin biosynthesis pathway. These LBGs are closely related to the accumulation of cyanidin derivatives (Fig. 5c). So, they are likely to play a major regulatory role in the pigmentation of lily petals.

*LvMYB5* and *LvMYB7* were clustered in subgroup 6 of the MYB gene family. These genes could regulate the expression of LBGs and biosynthesis of late anthocyanins [33, 78]. Similarly, the *MdMYB10* of *Malus,* clustered in subgroup 6, was positively correlated with anthocyanin accumulation and LBGs expression [42]. Overexpression of MYB-related TFs in tomato fruits leads to high expression of flavonoid related genes, such as *F3’H, F3’5’h, ANS,* and *UFGT,* leading to high accumulation of anthocyanins [79]. Research shows that *LhMYB12* regulates the pigmentation of anthocyanins in petals, spots, and ovaries, by inhibiting or enhancing the expression of anthocyanin biosynthesis genes [4, 27]. So, there may be multiple MYB genes that can synergistically regulate flower color formation in different tissues of plants.

## Conclusions

Anthocyanin biosynthesis is a complex pathway that is significant in flower coloration. The encoded enzyme genes involved in anthocyanin biosynthesis in flowers are active in flower pigmentation, but the relationship of anthocyanin accumulation with the different regions of petals is unclear. In Oriental hybrid lily cultivar ‘Vivian’, the top (pigmented) and bottom (un-pigmented) regions of the same petal show different color; however, the special mechanism is still unclear. In this study, we found that the morphologies in epidermal cells in different color regions of lily petal were different. To understand whether the cell morphological difference was related with metabolite biosynthesis and gene expression, metabonomics and transcriptome analysis were used here. Results of the metabonomics analysis indicated that cyanidin derivatives were accumulated in the main pigment, but flavanones were accumulated and anthocyanins were decreased in the un-pigmented regions of lily petal (X). This suggests that the biosynthesis of anthocyanins and flavanones might be involved in the regulation of lily flower color. Thus, the expression of these genes-related the biosynthesis of anthocyanins and flavanones was analyzed in the pigmented and un-pigmented regions of lily petal by RNA-Seq and qRT-PCR. Among these genes, the expression levels of *LvF3’H, LvDFR, LvANS, Lv3GT, LvMYB5* and *LvMYB7* were significantly different between the pigmented and un-pigmented regions of petals, and positively correlated with the accumulation of cyanidin derivatives. This suggests that these genes might regulate the biosynthesis of anthocyanins to affect the flower color formation in the pigmented and un-pigmented regions of petals in lily. It will help us to further understand the regulation network of lily petal pigmentation and cultivate different unique color species.

We identified and annotated a large number of unigenes, providing a good platform for lily genome research, and proposed a new perspective for studying the regulation mechanism of flower color related gene expression during the development of lily flowers. However, further validation is required at the cellular and transcriptional levels, to determine how the TF MYB gene regulates the genes to promote petal pigmentation in lily. The conclusion of this study provides a new direction for the study of flower color, allowing further study of the functions of key factors.

## Methods

### Plant materials and treatments

Lily cultivars were cultivated in a greenhouse (unheated and natural photoperiod) at the experimental farm of Shenyang Agricultural University, Liaoning province, China in 2018. Petals from different periods and different regions were used as plant materials, including: S1 – bud stage petals 20 days after bud formation; S2 – coloring bud petals 30 days after bud formation; blooming stage petals 40 days after bud formation, which was divided into S3 – the pigmented region in blooming stage petals, and X – the un-pigmented region in blooming stage petals. Four petal samples were collected with three biological replicates, then immediately frozen in liquid nitrogen for more than 30 min, and stored in a refrigerator at − 80 °C for further study.

### Scanning electron microscopy

Lily cultivars ‘Corvara’, ‘Table dance’, ‘Vestaro’ and ‘Vivian’ were oriental hybrid lily flowers. The petals of these lily were consisted of red/pink and white, and displayed a color gradient from pigmented to un-pigmented. So, we selected these cultivars to explore the difference of cell morphology. Then we selected one of them for further research and analysis.

Plant material was rinsed with distilled water, and a square sample of 5 mm × 5 mm was rapidly excised. Treated samples were fixed in 1 ml 2.5% glutaraldehyde buffer under vacuum. The sample was washed 3 times with a phosphate buffer of 1.5 ml pH 7.2, for 10 min each time, to remove the residual fixative on the surface. Next, the fixed tissues were dehydrated in an ascending aqueous ethyl alcohol series (30, 50, 70, 90, and 100%) in 1 mL additional volume. The ethanol was replaced in the cells with isoamyl acetate, 2 times, for 15 min each time. Lastly, the solvents in the samples were replaced with liquid carbon dioxide by a critical-point drying method [80]. The dry tepals were mounted on a specimen stub and sputter-coated with gold before examination using the Hitachi Regulus 8100 scanning electron microscope (SEM). The samples were observed and photographed by SEM.

### Metabolite identification and quantification

All samples were measured in random order using the ultra-performance liquid chromatography (Shim-pack UFLC SHIMADZU CBM30A, http://www.shimadzu.com.cn/) and tandem mass spectrometry (Applied Biosystems 6500 QTRAP, http://www.appliedbiosystems.com.cn/) (UPLC-MS/MS) system. Biological samples were vacuum freeze-dried and ground (30 Hz, 1.5 min) to powder using a grinding apparatus (MM 400, Retsch). A total of 100 mg powder was weighed and dissolved in 1.0 mL extract. The dissolved samples were vortexed three times during 4 °C refrigeration overnight to improve the extraction yield. After centrifugation (10,000×g, 10 min), the supernatant was extracted. The sample was filtered with a micropore filter (0.22 m pore size), and stored in a vial for LC-MS/MS analysis. The UPLC effluent was connected to an electrospray ionization (ESI)- triple quadrupole-linear ion trap-MS/MS system. The ESI source was set to positive ionization mode, the source temperature was held at 500 °C; the capillary voltage was 5500 V. The monitoring mode was set to multiple-reaction monitoring (MRM).

The identification of compounds detected by LC–MS was carried out based on a search of accurate masses of significant peak features against the online KEGG (http:// www.kegg.jp/) and HMDB (http://www.hmdb.ca) databases. A metabolite name was reported when the mass difference between observed and theoretical compounds was < 10 ppm. The identification and quantification of metabolites were carried out following Chen et al. [81]. Metabolites with significant differences in content were set with thresholds of variable importance in projection (VIP) ≥ 1 and fold change ≥2 or ≤ 0.5.

### RNA extraction

The total RNA of petal samples S1, S2, S3, and X were extracted by RNA plant plus (TIANGEN, Beijing, China) following the manufacturer’s instructions. The quality of purified RNA was initially evaluated on agarose gel and then quantified using a NanoDropTM spectrophotometer (Thermo Fisher Scientific, Inc.).

### RNA-Seq and annotation

The petal samples S1, S2, S3, and X were selected for transcriptome sequencing using Illumina Hiseq (Personal Biotechnology, Shanghai, China). The mRNA with polyA structure in the total RNA was enriched by Oligo (dT) magnetic beads. The mRNA was purified and fragmented. Then, the product was broken into short segments of 200–300 bp. After that, the double-stranded cDNA was synthesized. cDNA synthesis was performed to construct the libraries. The library quality assessment was conducted on the Agilent 2100 Bioanalyzer system and the library was sequenced on Illumina Hiseq platform with generating paired-end reads.

Sequencing data were processed to remove adaptor and low-quality reads. All the downstream analyses were based on clean data with high quality. De novo assembly of the transcriptome was performed to gain transcript sequences using the Trinity software with default parameters and no reference sequence [82]. The transcripts were clustered by hierarchical cluster analysis [83] and then the longest transcript sequence was selected from each cluster as unigene. Only high-quality reads (clean reads) were used for statistical analysis to ensure the accuracy and reliability of the RNA-sequencing data.

The functional annotation information for differentially expressed genes (DEGs) was obtained using the following databases: NR (NCBI nonredundant protein sequences, ftp://ftp.ncbi.nih.gov/blast/db/), Pfam (http://pfam.xfam.org/), KOG/COG/eggNOG (Clusters of Orthologous Groups of proteins, ftp://ftp.ncbi.nih.gov/pub/COG/COGhttp://eggnogdb.embl.de/), Swiss-Prot (http://www.uniprot.org/), KEGG (Kyoto Encyclopedia of Genes and Genomes, http://www.genome.jp/kegg/), and GO (Gene Ontology, http://www.geneontology.org/) [84].

The FPKM values of anthocyanin biosynthetic genes were log2 transformed (in order to avoid the minus value after transformation, we added ‘1’ to every FPKM values before log2 transformation), mean centered, then a row standardization was performed. The heatmap generated by the heatmap package in R software.

### qRT-PCR analysis

The first strand of cDNA was synthesized using the HiScript 1st Strand cDNA Synthesis Kit (Vazyme, Nanjing, China). AceQ qPCR SYBR Green Master Mix (without ROX) (Vazyme, Nanjing, China) was used to intercalate the SYBR Green into amplified products. The 10 μl reaction solution contained 0.5 μl of each gene-specific primer and 2 μl of ten times diluted first-strand cDNA. Signals were monitored using a BIO-RAD CFX 96 touch qRT-PCR system (Bio-Red Laboratories, Inc., Hercules, CA, USA). The amount of actin mRNA in each sample was determined and used to normalize the amounts of mRNA of the target genes. The following cycling parameters were used: 1 hold at 95 °C for 5 min, 40 cycles at 95 °C (10 s) and 60 °C (25 s), 1 hold at 95 °C (15 s), 60 °C (60 s), 95 °C (30 s), 95 °C (15 s) to fusion curve collection. Primer specificity was confirmed by a melting curve analysis and agarose gel electrophoresis of the qRT-PCR products. The relative expression of genes was calculated based on the 2^-ΔΔCt^ method, where ΔCt = Ct (target gene)-Ct (actin). Relative fold-change values of three biological replicates were used to calculate mean values and standard errors [85]. The transcript levels are presented as the mean ± standard error of the mean. Statistical analysis including variance and significant difference were conducted using SPSS 16.0 (SPSS Inc., Chicago, IL, USA). The different letters indicate statistically significant differences among samples. The primers used in the experiment are listed in Supplementary table (Additional file 13: Table S6).

The cDNA was diluted 10 times by gradient, with a total of 5 gradients of 0.1 0.01 0.001 0.0001 0.00001. Then the series of diluents were used as templates for qRT-PCR with different primers. The Ct values were obtained to draw the standard curves for individual primers. The regression coefficient of each primer standard curve was above 0.9, indicating a good correlation between Ct value and template dilution ratio. The primer efficiency ranged from 90 to 105%, which were conform to the requirement of qRT-PCR.

### Correlation analysis

Correlation analysis of anthocyanin structural genes and transcription factors was performed to obtain the main putative genes related to anthocyanin biosynthesis. Pearson correlation analysis was carried out between TFs and structural genes, and between genes and metabolites using the ‘correlate’ function in SPSS 16.0 software. Then, we constructed a network using Cytoscape (version 3.6.1, the Institute of Systems Biology, Seattle, Washington, USA) for visualization [86].

### Phylogenetic analysis

The phylogenetic analysis based on the amino acid sequences was performed in MEGA (version 5.0, the laboratory at the Pennsylvania State University, St Collie, PA, USA) and the Maximum Likelihood method was used with 1000 bootstrap replicates [87].

## Supplementary information

**Additional file 1: Figure S1.** Electron microscopic observation of the epidermal cell structure of lily petals during the blooming stage. (a): The white flower and petal of Oriental hybrid lily; (b): Lily cultivar ‘Vivian’ flower and petal; Bar = 10 mm (c): the morphology of epidermal cells in different regions of lily petals with magnifications of 200 times (left, Bar = 200 μm) and 400 times (right, Bar = 100 μm). The samples were observed and photographed by SEM.

**Additional file 2: Figure S2.** KEGG analysis of the differentially accumulated metabolites in X vs. S3.

**Additional file 3: Figure S3.** KEGG analysis of the differentially accumulated metabolites in S1 vs. S3.

**Additional file 4: Figure S4.** Gene ontology enrichment analysis of the unigene.

**Additional file 5: Figure S5.** The differentially expressed genes (DEGs) in different stages of flower development. (a): Venn diagram of DEGs in flower development. (b): KEGG Pathway Enrichment in bud petals vs. coloring petals. (c): KEGG Pathway Enrichment in coloring petals vs. pigmented petals. (d): KEGG Pathway Enrichment in bud petals vs. pigmented petals.

**Additional file 6: Figure S6.** Evolutionary tree analysis of transcription factors. (a): MYB evolution analysis of lily and *Arabidopsis thaliana*; (b): Evolutionary tree analysis of the conserved motif structure of the MYB gene associated with anthocyanin synthesis (c): bHLH evolution analysis of lily and *Arabidopsis thaliana*; (d): Evolutionary tree analysis of the conserved motif structure of the bHLH gene associated with anthocyanin synthesis.

**Additional file 7: Figure S7.** Evolutionary tree analysis of WDR in lily and plant.

**Additional file 8: Table S1.** All the metabolites of lily petals detected by widely-targeted metabolomics method.

**Additional file 9: Table S2.** Statistics of RNA-Seq data.

**Additional file 10: Table S3.** Statistics of sequence.

**Additional file 11: Table S4.** Summary for the annotation of unigenes.

**Additional file 12: Table S5.** The pearson correlation of transcriptome data and qRT-PCR.

**Additional file 13: Table S6.** The primers information of 8 genes in this study.

**Additional file 14: Figure S8.** Heatmap of gene expression involved in cell morphology in lily cultivar ‘Vivian’ petals. Each colored cell represents the average log_2_(FPKM) value of each sample gene, then performed rows cluster.

## Data Availability

The datasets generated and analyzed during the current study are available at NCBI project PRJNA649743. Any reasonable requests are available from the corresponding author.

## Abbreviations

DEG: Differentially Expressed Gene
DHK: Dihydrokaempferol
DHM: Dihydromyricetin
DHQ: Dihydroquercetin
GO: Gene ontology
KEGG: Kyoto Encyclopedia of Genes and Genomes
UPLC-MS/MS: Ultra-performance liquid chromatography and tandem mass spectrometry
LBG: Late biosynthetic genes
EBG: Early biosynthetic genes
TFs: Transcription factors
