# Supplementary figures and images for: Integrated metabolic profiling and transcriptome analysis of pigment accumulation in diverse petal tissues in the lily cultivar ‘Vivian’
Source: BMC Plant Biol. 2020 Sep 29;20:446. doi: 10.1186/s12870-020-02658-z (PMC7526134; doi:10.1186/s12870-020-02658-z)

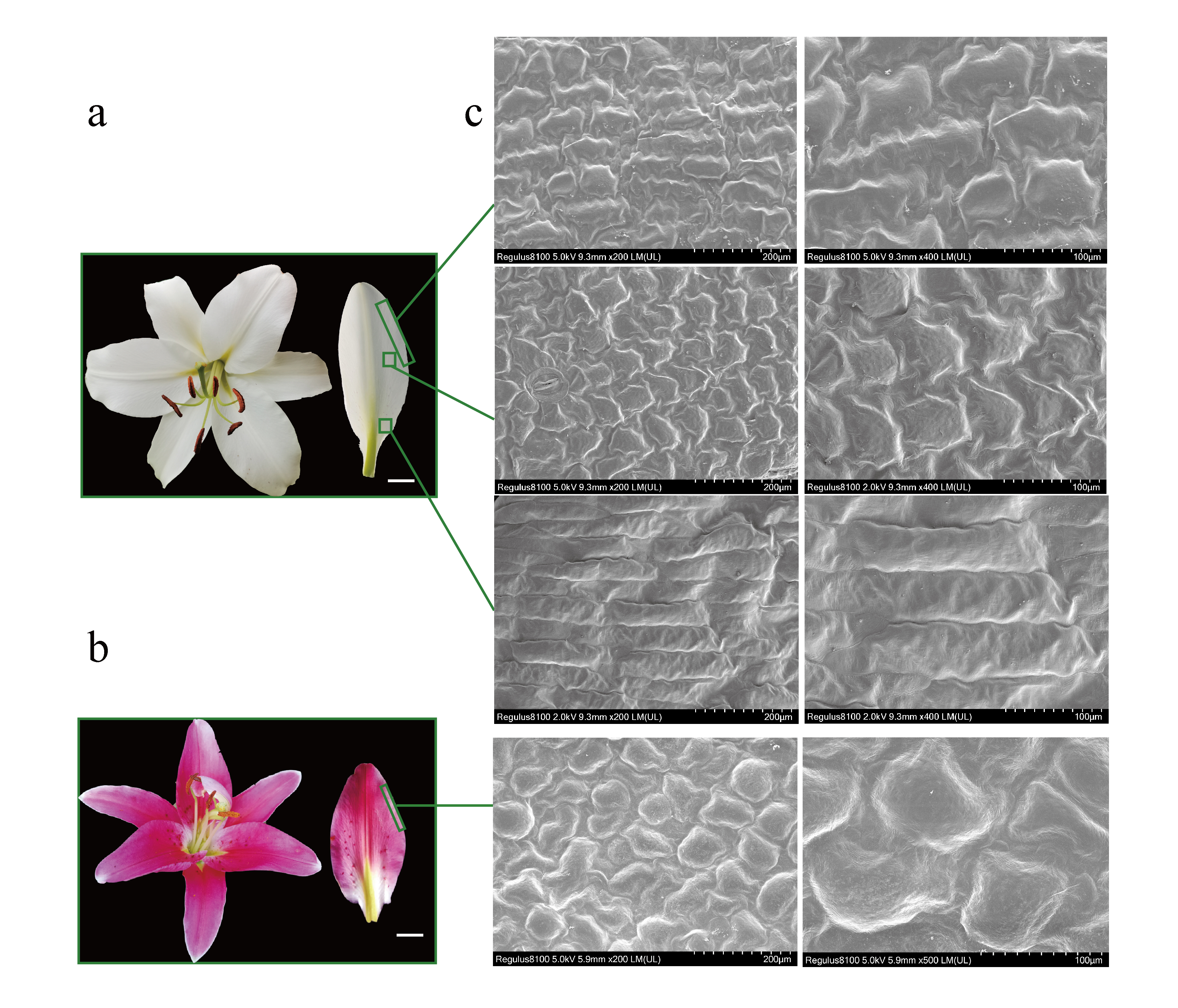

Supplement: Supplementary file 1 — Additional file 1: Figure S1. Electron microscopic observation of the epidermal cell structure of lily petals during the blooming stage. (a): The white flower and petal of Oriental hybrid lily; (b): Lily cultivar ‘Vivian’ flower and petal; Bar = 10 mm (c): the morphology of epidermal cells in different regions of lily petals with magnifications of 200 times (left, Bar = 200 μm) and 400 times (right, Bar = 100 μm). The samples were observed and photographed by SEM. [file 12870_2020_2658_MOESM1_ESM.jpg]

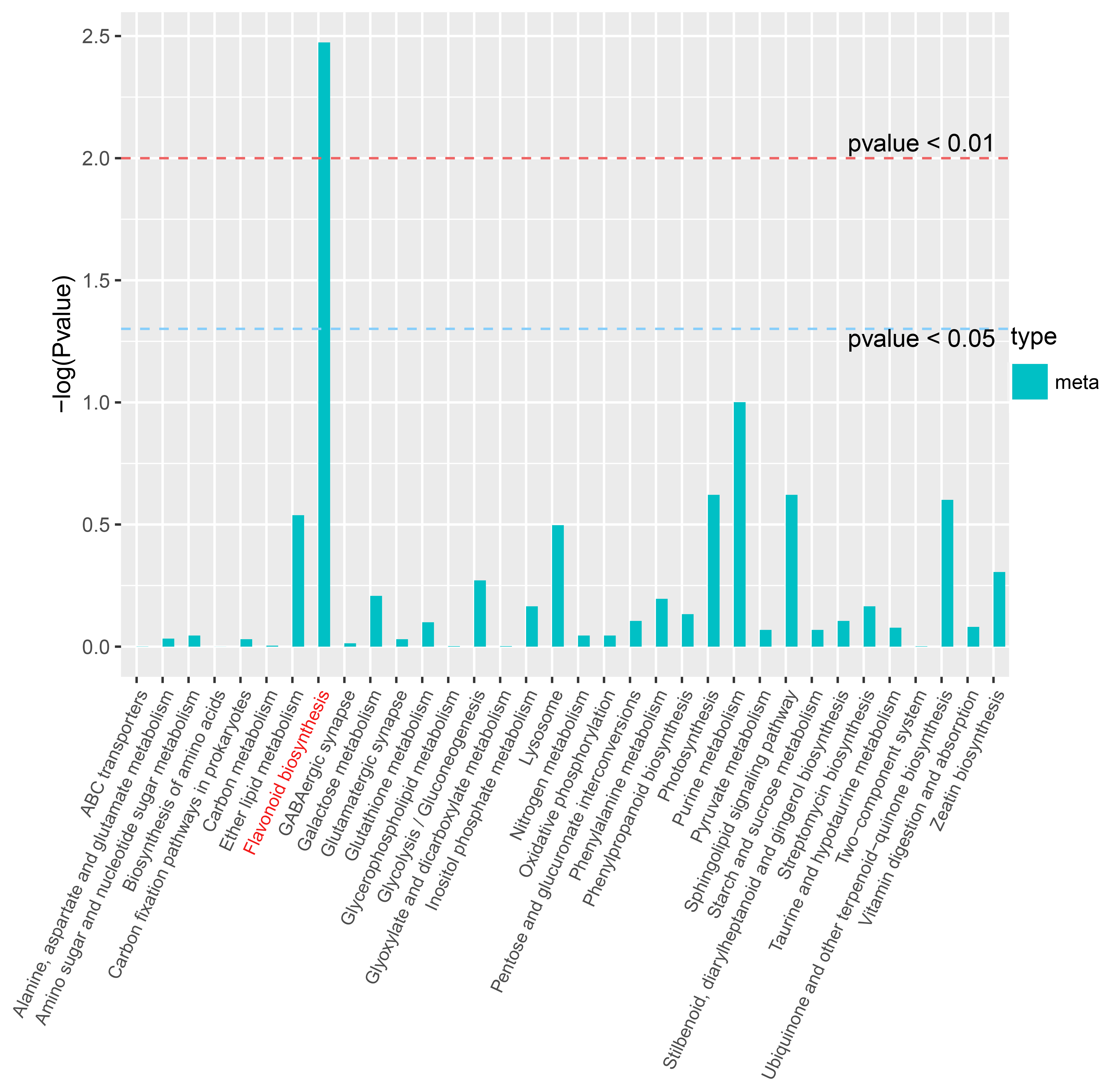

Supplement: Supplementary file 2 — Additional file 2: Figure S2. KEGG analysis of the differentially accumulated metabolites in X vs. S3. [file 12870_2020_2658_MOESM2_ESM.jpg]

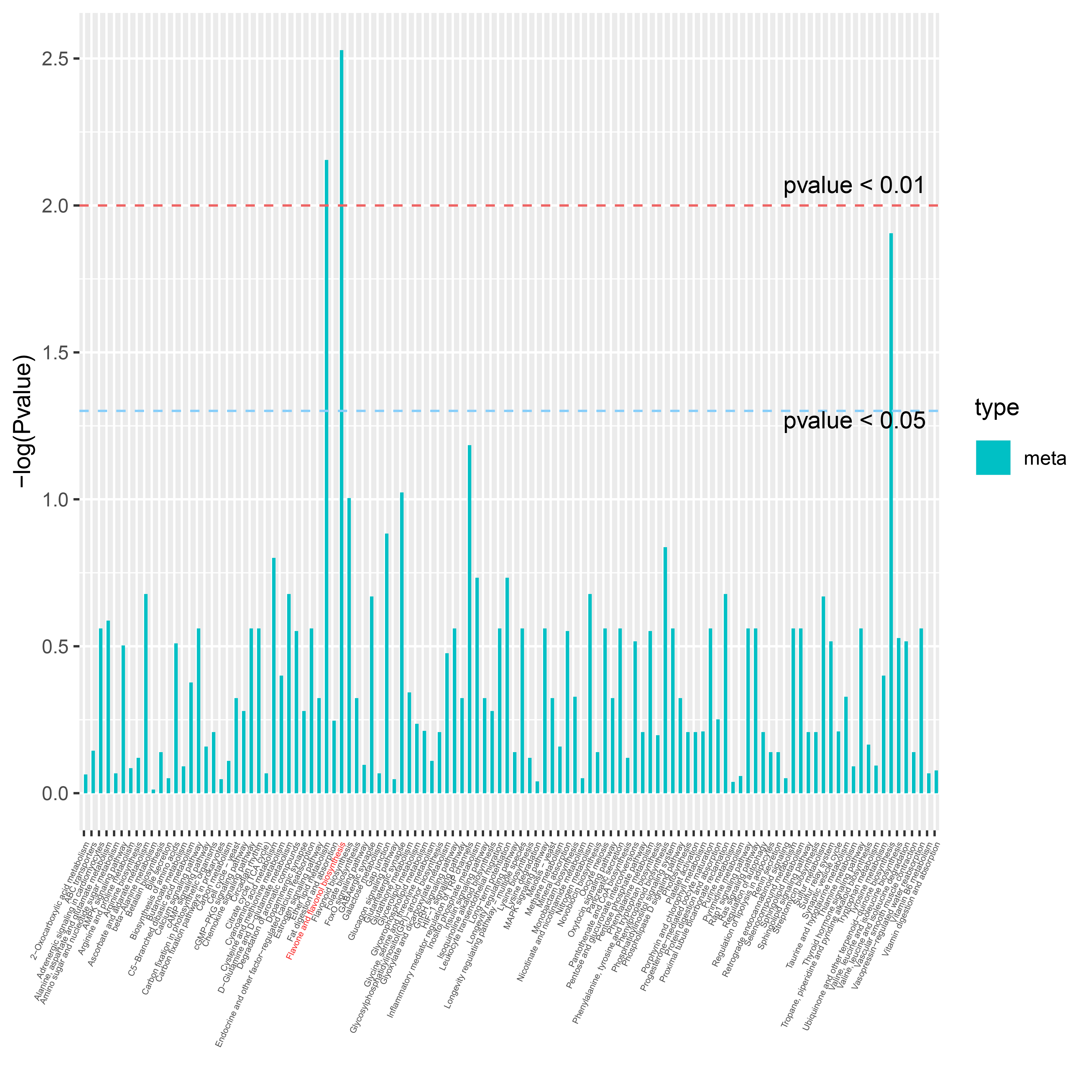

Supplement: Supplementary file 3 — Additional file 3: Figure S3. KEGG analysis of the differentially accumulated metabolites in S1 vs. S3. [file 12870_2020_2658_MOESM3_ESM.jpg]

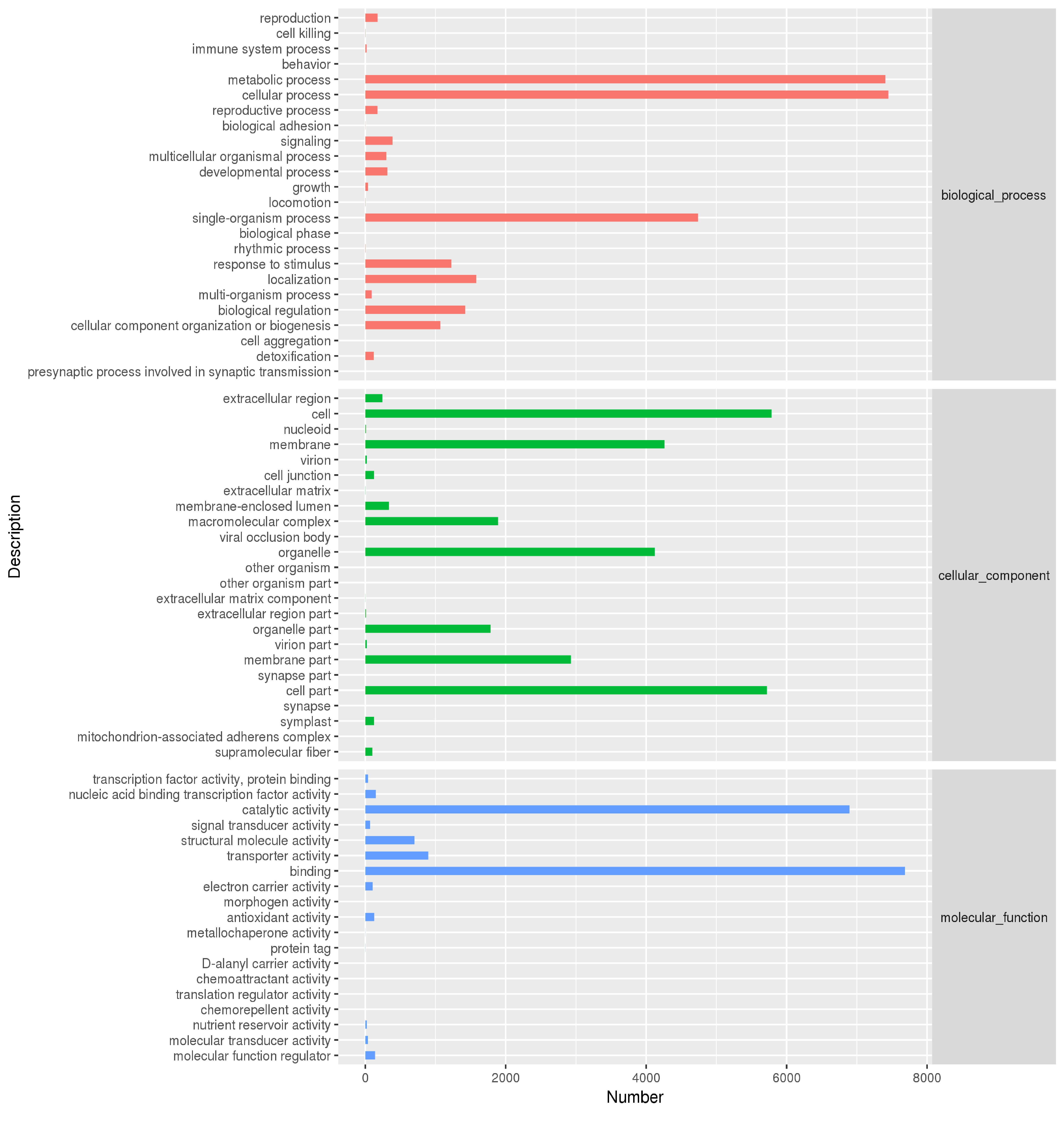

Supplement: Supplementary file 4 — Additional file 4: Figure S4. Gene ontology enrichment analysis of the unigene. [file 12870_2020_2658_MOESM4_ESM.jpg]

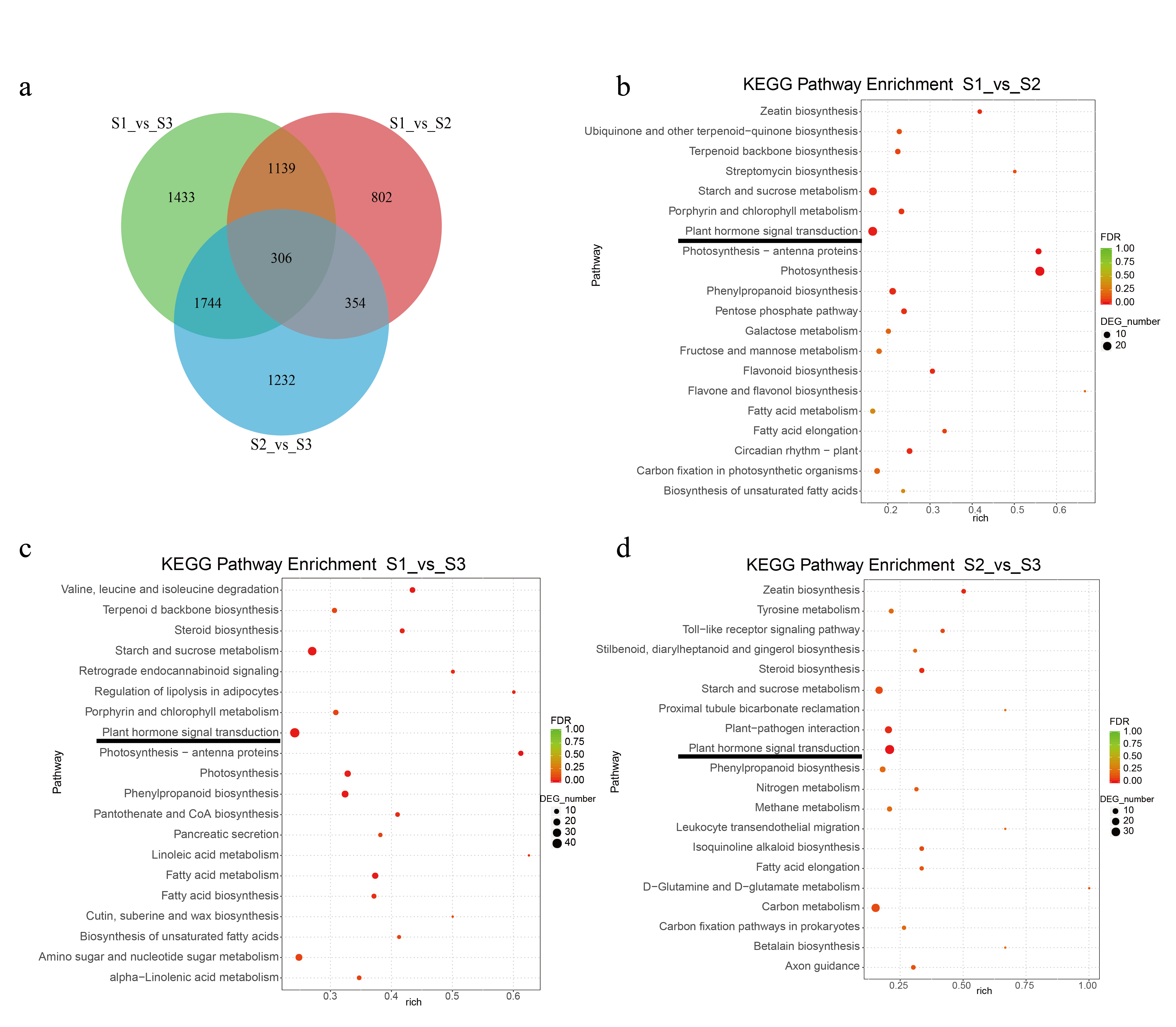

Supplement: Supplementary file 5 — Additional file 5: Figure S5. The differentially expressed genes (DEGs) in different stages of flower development. (a): Venn diagram of DEGs in flower development. (b): KEGG Pathway Enrichment in bud petals vs. coloring petals. (c): KEGG Pathway Enrichment in coloring petals vs. pigmented petals. (d): KEGG Pathway Enrichment in bud petals vs. pigmented petals. [file 12870_2020_2658_MOESM5_ESM.jpg]

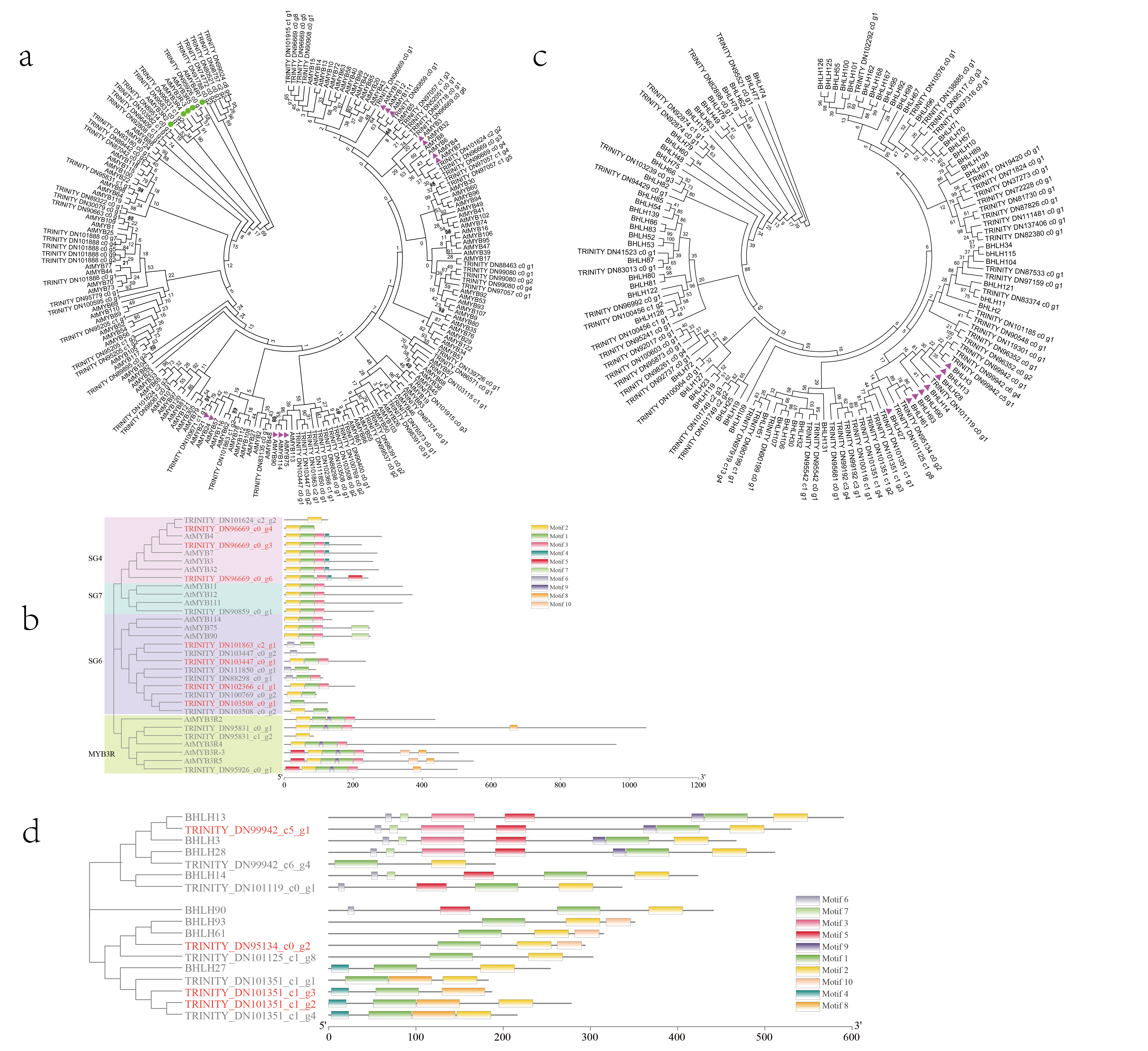

Supplement: Supplementary file 6 — Additional file 6: Figure S6. Evolutionary tree analysis of transcription factors. (a): MYB evolution analysis of lily and Arabidopsis thaliana; (b): Evolutionary tree analysis of the conserved motif structure of the MYB gene associated with anthocyanin synthesis (c): bHLH evolution analysis of lily and Arabidopsis thaliana; (d): Evolutionary tree analysis of the conserved motif structure of the bHLH gene associated with anthocyanin synthesis. [file 12870_2020_2658_MOESM6_ESM.jpg]

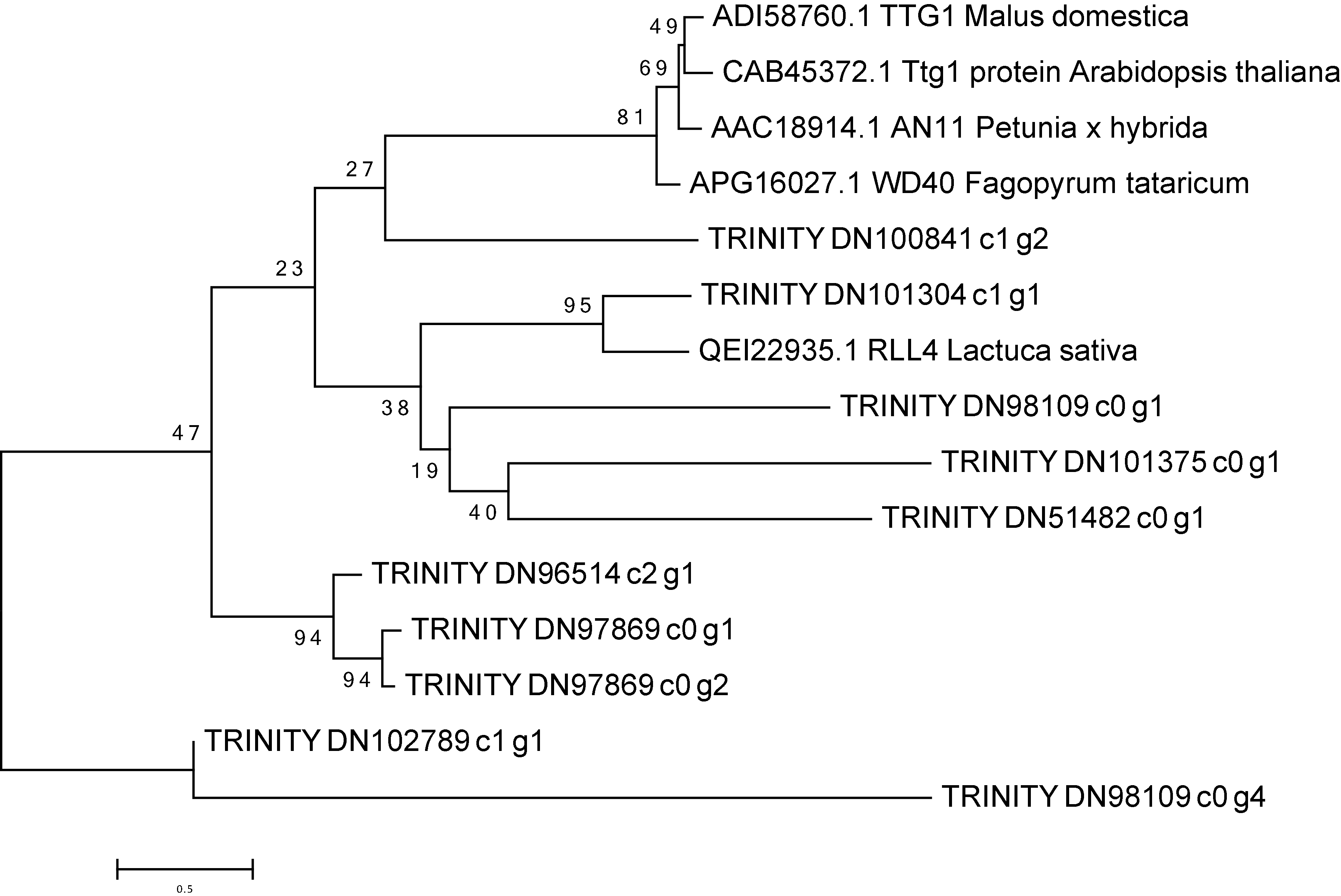

Supplement: Supplementary file 7 — Additional file 7: Figure S7. Evolutionary tree analysis of WDR in lily and plant. [file 12870_2020_2658_MOESM7_ESM.jpg]

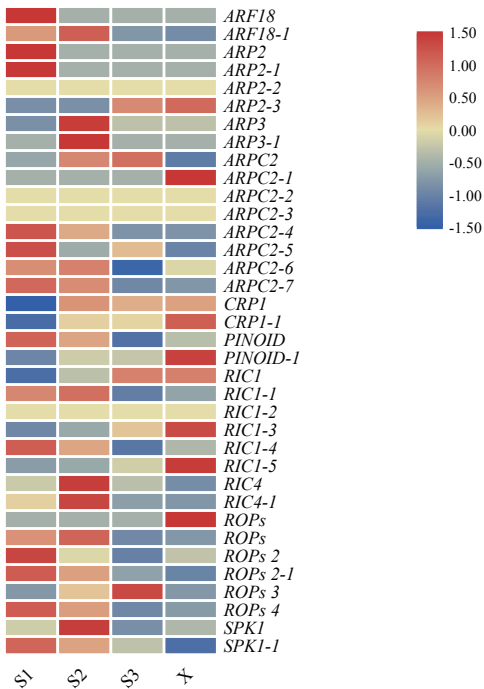

Supplement: Supplementary file 14 — Additional file 14: Figure S8. Heatmap of gene expression involved in cell morphology in lily cultivar ‘Vivian’ petals. Each colored cell represents the average log2(FPKM) value of each sample gene, then performed rows cluster. [file 12870_2020_2658_MOESM14_ESM.pdf]
