# Supplementary material for: Integrated metabolic profiling and transcriptome analysis of pigment accumulation in diverse petal tissues in the lily cultivar ‘Vivian’
Source: BMC Plant Biol. 2020 Sep 29;20:446. doi: 10.1186/s12870-020-02658-z (PMC7526134; doi:10.1186/s12870-020-02658-z)
Supplement: Supplementary file 12 — Additional file 12: Table S5. The pearson correlation of transcriptome data and qRT-PCR. [file 12870_2020_2658_MOESM12_ESM.docx]

**Table S5**. The pearson correlation of transcriptome data and qRT-PCR.

| Gene name | *LvMYB5* | *LvMYB7* | *LvCHS* | *LvF3’H* | *LvDFR* | *LvANS* | *Lv3GT* |
| --- | --- | --- | --- | --- | --- | --- | --- |
| Pearson correlation (r) | 0.8205 | 0.8197 | 0.9241 | 0.8025 | 0.8790 | 0.8004 | 0.8006 |
