# Supplementary material for: Integrated metabolic profiling and transcriptome analysis of pigment accumulation in diverse petal tissues in the lily cultivar ‘Vivian’
Source: BMC Plant Biol. 2020 Sep 29;20:446. doi: 10.1186/s12870-020-02658-z (PMC7526134; doi:10.1186/s12870-020-02658-z)
Supplement: Supplementary file 13 — Additional file 13: Table S6. The primers information of 8 genes in this study. [file 12870_2020_2658_MOESM13_ESM.docx]

**Table S6.** The primers information of 8 genes in this study

| Gene | Forward sequence (5'-3') | Reverse sequence (5'-3') | PCR Producs (bp) | primer efficiency（%） | Regression coefficient (R^2^) |
| --- | --- | --- | --- | --- | --- |
| *Actin* | GTCCATCCATCGTCCACAG | CCTCAACAAGCCACCTACC | 180bp | 98.99 | 0.9985 |
| *LvDFR* | GGGTCCTTTCATCACCTCAAC | CCACTCGCTTCTGGATTCTCA | 165bp | 96.31 | 0.9946 |
| *LvANS* | CCAAATGTCCGCAACCTGA | GCCAATGTGGACGAGAAGC | 164bp | 99.23 | 0.9962 |
| *Lv3GT* | GGCAACCTTGACTCCGTCTT | GGGGATTGTTGGGTGAGGA | 182bp | 96.83 | 0.9981 |
| *LvCHS* | CTCCGTCAACCGCCTCAT | TGATCTCCGAGCAGACCACTA | 125bp | 98.39 | 0.9914 |
| *LvF3’H* | CACAGCAGGGACAGACACCA | GGCACTTAGGAAGGGGAGG | 166bp | 99.61 | 0.9959 |
| *LvMYB5* | AGTGAAGTGGAGCCGTGTT | TGTGAAGCCTGATGATGAGGT | 146bp | 97.36 | 0.9948 |
| *LvMYB7* | CGACGGCGATGGCTCAACTA | GCTGGGTTACAGGTTGTGGTCT | 153bp | 96.82 | 0.9910 |
